# Genome size data for eight endemic plant species from the Nanda Devi Biosphere Reserve (Western Himalaya)

**DOI:** 10.1016/j.dib.2024.110450

**Published:** 2024-04-18

**Authors:** Jaya Arora, Suman Lakhanpaul, Kumar Manish, Maharaj K. Pandit

**Affiliations:** a Department of Environmental Studies, University of Delhi, Delhi, India; b Centre for Interdisciplinary Studies of Mountain & Hill Environment, University of Delhi, Delhi, India; c Department of Botany, University of Delhi, Delhi, India; d Jindal School of Environment & Sustainability, O.P. Jindal Global University, Sonipat, Haryana, India; e National University of Singapore, Residential College 4, 6 College Avenue East, Singapore

**Keywords:** Biodiversity, Ecology, Flow cytometry, Genomics

## Abstract

The Himalaya harbors a large number of plant endemics but information on their genome size is largely lacking. This study aims to fulfill this gap by reporting genome sizes for 8 endemic Himalayan plant species (*Impatiens devendrae* Pusalkar, *Impatiens scabrida* DC., *Impatiens sulcata* Wall., *Geranium robertianum* L., *Geranium wallichianum* D.Don ex Sweet, *Thalictrum cultratum* Wall., *Thalictrum elegans* Wall. ex Royle, *Thalictrum foliolosum* DC.) from the Western Indian Himalayan state of Uttarakhand. The study involved collecting leaf tissues from each of the 8 plant species, chopping, staining and estimating nuclear DNA content using CyFlow Cube 8 flow cytometer with 532 nm laser light source and an orange-red fluorescence emission (>590 nm). The CyFlow Cube 13 programme was utilised to obtain the median fluorescence value from PI-stained G0/G1 (quiescent phase/first growth phase) nuclei, devoid of cellular debris. The DNA 2C value of each sample was then estimated by comparing the median fluorescence intensity values of both sample and standard (*Solanum lycopersicum* L.) using the standard scientific formula. The highest DNA 2C-values were observed in *Geranium*, which ranged from 5.29 ± 0.02 pg to 2.49 ± 0.02 pg. The genome size of *Impatiens* species varied from 1.49 ± 0.08 pg to 3.14 ± 0.04 pg while the three species of genus *Thalictrum* had nearly similar genome sizes varying between 1.53 ± 0.01 pg to 1.96 ± 0.06 pg. The coefficient of variation among nuclei varied from 3.52 % to 5.38 % with 103 to 1811 numbers of stained nuclei. The results and framework presented in the current study can serve as a template for future studies that attempt to estimate the genome sizes of endemic plant species in the Himalaya, a global biodiversity epicentre and one of the least studied biodiversity hotspots of the world.

Specifications Table

**Table:** 

Subject	Molecular Biology
Specific subject area	Genome size estimation
Type of data	Raw, Analyzed and Filtered
Data collection	To determine the genome size of 8 plant species, fresh leaf tissues were harvested from the young leaves in the field and transported to the laboratory and further stored at 4 °C. Assays were conducted within 4–5 days of collection. Sample acquisition was done using CyFlow Cube 8 flow cytometer (Sysmex Partec GmbH, Germany), equipped with 532 nm laser light source and an orange-red fluorescence emission (>590 nm). Raw data was acquired using the CyFlow Cube 13 software and FCS express software (7.04.0018) was used for data representation. To minimize experimental error, tissue-specific nuclear DNA content was estimated using three technical replicates of both test samples and internal control.
Data source location	**Institution**: CISMHE's Himalaya Lab and Central Instrumentation Facility, Department of Botany, University of Delhi, Delhi, India. **Country**: India **Field Location**: Valley of Flowers National Park in Nanda Devi Biosphere Reserve of Western Himalaya, Chamoli District, Uttarakhand, India. **Geographical Coordinates**: 31°04′ N to 30°06′ N latitude and 79°13′ E to 80°17′ E longitude (see Fig. 1).
Data accessibility	**Repository name**: Mendeley **Data identification number**: 10.17632/w54dhkykr3.2 **Direct URL to data**: https://data.mendeley.com/datasets/w54dhkykr3/2

## 1 Value of the Data

• There remains a wide gap between the number of plant species described in the regional floras and information on their genome sizes in the Himalayan environments as genome sizes of merely 0.01 % of plant species are available in the Plant DNA C-value database (https://cvalues.science.kew.org/). The results of genome size from the present study would bridge a significant gap in the genome size data representation of endemic Himalayan plant species in the literature.
• The information on the genome size of plant species provides crucial information on their conservation status as scientific reports indicate that species with smaller genome sizes tend to be invasive, while those with larger genome sizes are disproportionately rare or endangered.
• The data on genome sizes can indicate the habitat preferences of plant species. Generally, species with large genome sizes are not selected in extreme environmental conditions such as high elevations with extreme weather conditions and short growth cycles.
• The protocols and genome size data reported in the present study can serve as a template for future eco-genomic studies in the Himalaya.

## 2 Background

Application of genome size data in ecological studies includes understanding the correlation between genome size and biological traits [1,2]. Recent studies have shown that genome size is an important genetic trait that indicates the conservation status of a plant species [2]. Taxa with large genome sizes tend to be more prevalent among rare/endangered plants whereas smaller genomes are associated with invasiveness [2]. While numerous investigations have been carried out on the cytogenetic profiles of the Himalayan plant populations, studies on endemic Himalayan plant species are lacking [3]. The reports of the plant genome sizes from the Himalaya are mostly confined to the Eastern Himalayan states such as Assam, Arunachal Pradesh and Meghalaya [4]. *Dendrobium fimbriatum* Hook. (DNA 2C= 6.2 pg), was the only endemic orchid from the Western Himalayan state Uttarakhand, whose genome size was reported [5]. Importantly, basic genomic attributes of the Himalayan flora remain largely unexplored. The urgency and importance of generating chromosome number and genome size data is particularly crucial for data-deficient regions of the world [6,7]. More plant species need to be investigated for these traits, reported and data generation must occur at a quicker pace. This study is an attempt in this direction.

## 3 Data Description

**Fig. 1:**
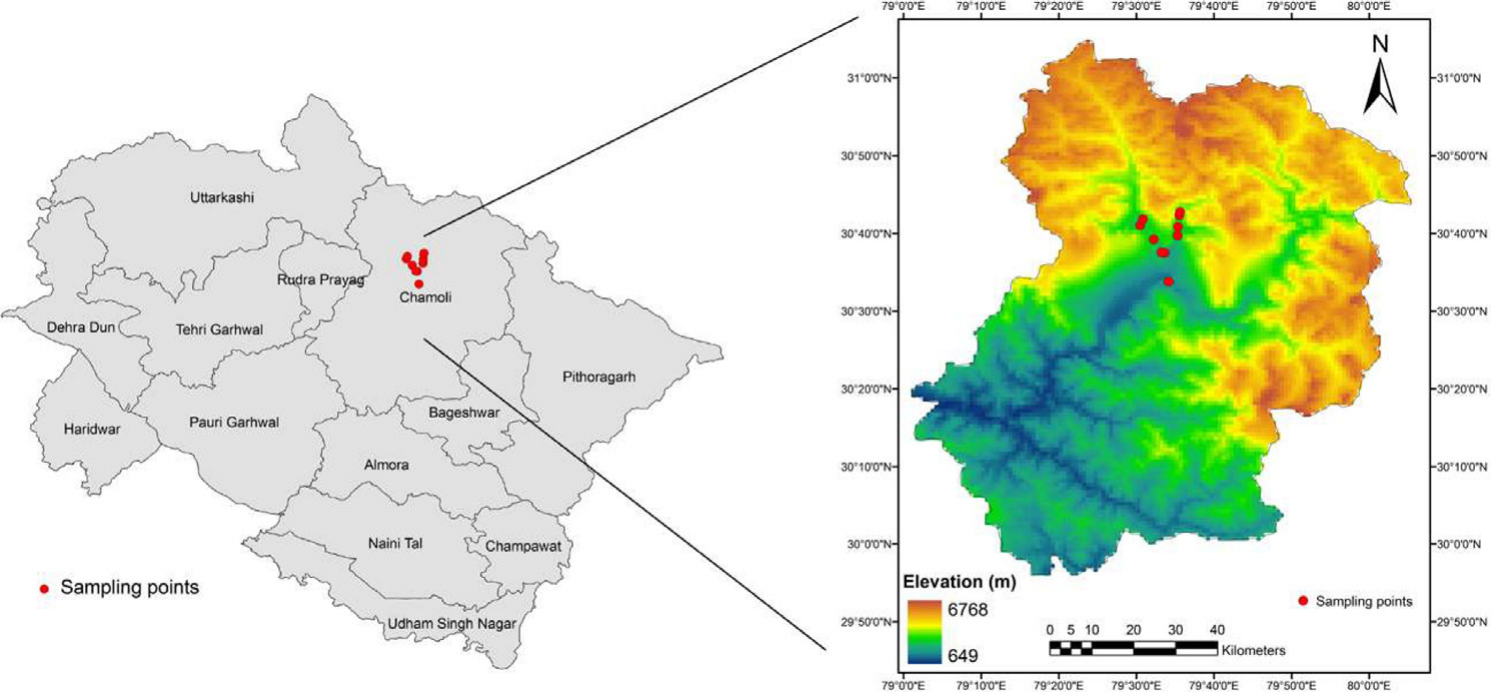
Map depicting the sampling locations of the present study. A total of 10 locations were sampled along an elevational gradient of 1430–3386 m in the Nanda Devi Biosphere Reserve of Uttarakhand (India).

**Fig. 2:**
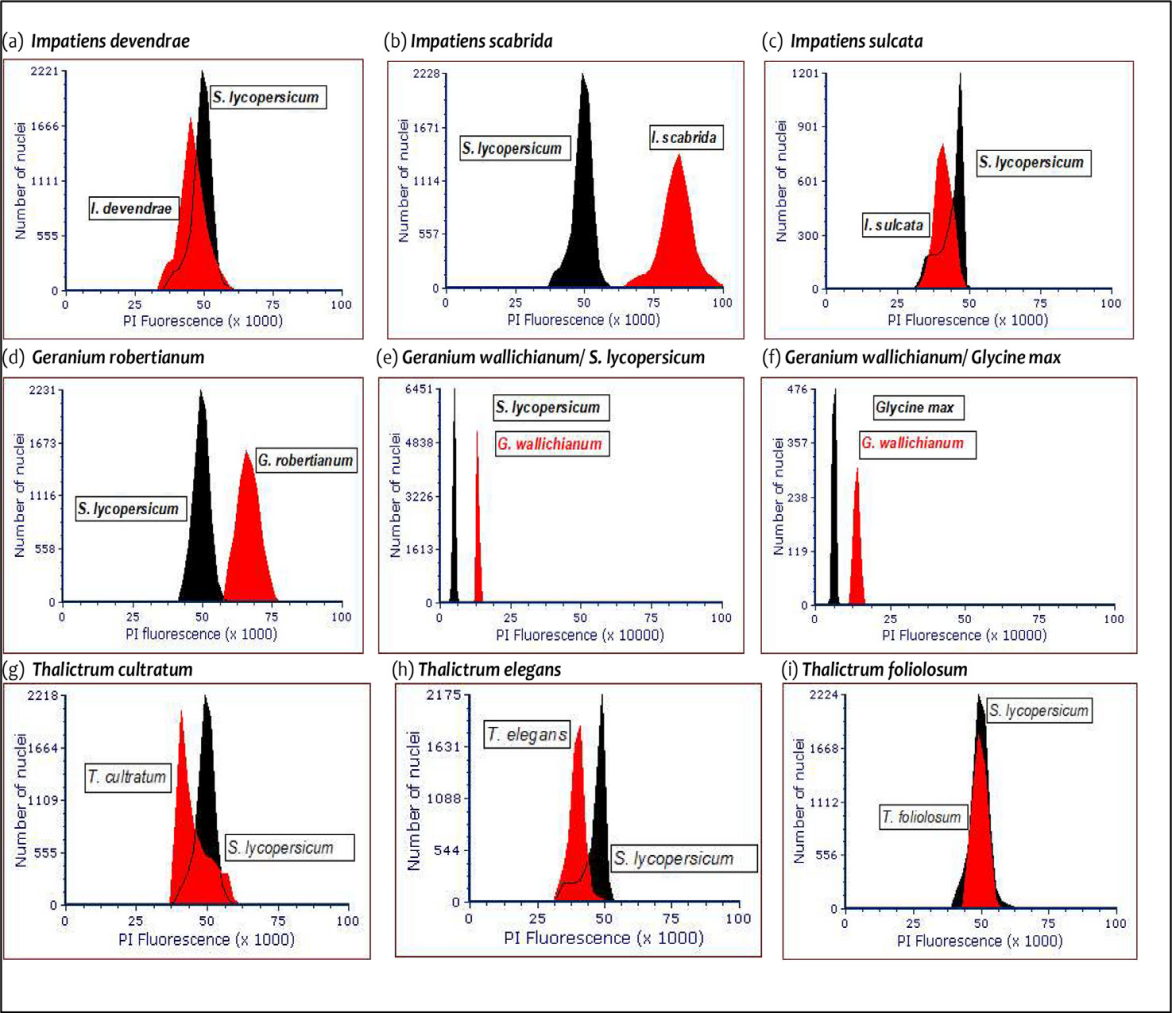
(a-i) Histograms of PI fluorescence intensity in eight Himalayan high-elevation taxa for genome size estimations using *Solanum lycopersicum* L. as an internal standard.

The dataset presented here consists of one figure and one table. Fig. 2 illustrates the histograms of propidium iodide (PI) fluorescence intensity in eight Himalayan endemic species for genome size estimations, with *Solanum lycopersicum* L. as an internal standard. The DNA 2C-values of the eight species investigated here are the first-ever reports for these taxa except for *Geranium robertianum* L. A recent report suggested the DNA 2C-value of *Geranium robertianum* L. to be 2.53 pg and chromosome number as 32 (2n = 32) [3,8]. Among the taxa investigated in this study, the highest DNA 2C-values were observed in *Geranium*, which ranged from 5.29 ± 0.02 pg to 2.49 ± 0.02 pg. The genome size of *Impatiens* species varied from 1.49 ± 0.08 pg to 3.14 ± 0.04 pg while the three species of genus *Thalictrum* had nearly similar genome sizes varying between 1.53 ± 0.01 pg to 1.96 ± 0.06 pg. The coefficient of variation among nuclei varied from 3.52 % to 5.38 % with 103 to 1811 numbers of stained nuclei Table 1. Overall, G1 peak of both the standard (*S. lycopersicum* L.) and sample at the same gain (400 V) appeared in nearby channels. Also, the 2C peaks of the sample species and the internal reference were positioned at channel number around 50 K in all the species (Fig. 1 a-i) except *Geranium wallichianum* D.Don ex Sweet*,* where the nuclear DNA content of the sample and the standards (*S. lycopersicum* L. and *Glycine max* (L.) Merr.) differed almost two-fold. The detailed results of flow cytometry (both as raw and processed FCS files) for each of the 8 species are available online in the Mendeley database (https://data.mendeley.com/datasets/w54dhkykr3/2) [9].

**Table 1:** Flow cytometric values of samples along with internal standard used for genome size estimation.

Sl.No.	Species Name	X-median value	Counts	CV%	Calculated 2C DNA value (pg)	Reported range of the genus: 2C DNA content (pg)
Standard	*Solanum lycopersicum* ‘Stupicke polni rane’	49,835	4226	3.52	**–**	**–**
1	*Impatiens devendrae* Pusalkar	45,739	1810	4.47	1.8 ± 0.05	0.80–6.51
2	*Impatiens scabrida* DC.	79,872	1109	4.9	3.14 ± 0.04	
3	*Impatiens sulcata* Wall.	37,888	1185	4.02	1.49 ± 0.08	
4	*Geranium robertianum* L.	63,329	103	4.82	2.49 ± 0.02	1.43–8.38
5	*Geranium wallichianum* D.Don ex Sweet	134,613	367	5.38	5.29 ± 0.02	
6	*Thalictrum cultratum* Wall.	38,844	1811	5.14	1.53 ± 0.01	0.49–7.48
7	*Thalictrum elegans* Wall. ex Royle	40,179	1138	5.1	1.58 ± 0.06	
8	*Thalictrum foliolosum* DC.	49,835	1490	5.18	1.96 ± 0.06

## 4 Experimental Design, Materials and Methods

**Fig. 3:**
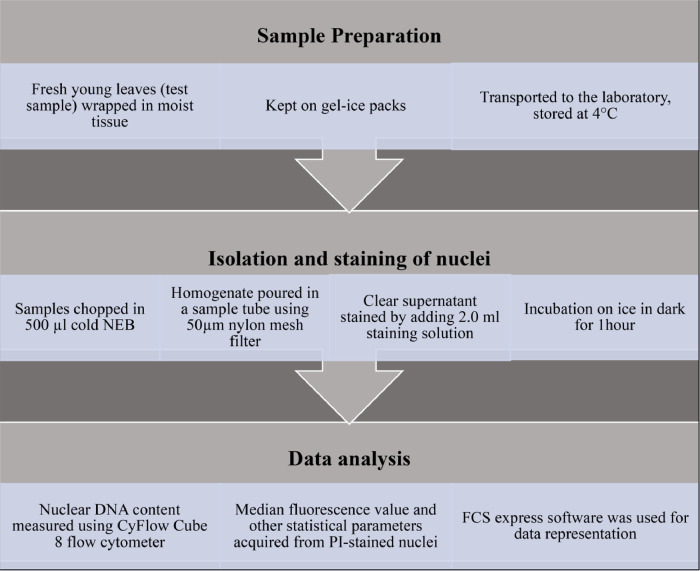
Flow chart describing the experimental procedure used in this study.

Depending on the abundance and availability of the plant material (young actively growing leaf samples), one to ten individuals of each of the eight plant species namely *Impatiens devendrae* Pusalkar, *Impatiens scabrida* DC., *Impatiens sulcata* Wall., *Geranium robertianum* L., *Geranium wallichianum* D.Don ex Sweet, *Thalictrum cultratum* Wall., *Thalictrum elegans* Wall. ex Royle, *Thalictrum foliolosum* DC. were sampled across 10 sampling sites in the Nanda Devi Biosphere Reserve in the Chamoli district of Uttarakhand (Fig. 1). Necessary permissions were obtained from the Department of Forests and Wildlife, Uttarakhand (Permission Number: 159/5–6 dated 21 July, 2016) for conducting the field investigations. Fresh leaf tissues were harvested from young leaves wrapped in moist tissue paper and kept on ice packs till they were transported to the laboratory and were further stored at 4 °C (Fig. 3). Assays were conducted within 4–5 days of collection because the study site was at a distant place from the laboratory. To minimize experimental error, tissue-specific nuclear DNA content was estimated using three technical replicates of both test-sample and internal-control (standard reference material). *Solanum lycopersicum* cv. “Stupicke polni rane” (1.96 pg/2C DNA) was used as an internal standard. Furthermore, *Glyci*ne *max* Merr. cv. Polanka’46 (2.5 pg/2C DNA) was used as the second reference standard to validate the unexpected results of this study. Seeds of the reference materials used as internal standards were cordially supplied by Dr. Jaroslav Doležel from the Experimental Institute of Botany, Czech Republic. These and experimental materials were germinated and grown in a growth chamber at 22 °C ± 2 °C with a relative humidity of 55 % ± 5 % under continuous white fluorescent light intensity of 60–90 µmol/m^2^. Nuclei from young and actively growing seedling leaves were extracted using Partec CyStain PI Absolute P kit: Sysmex, product number 05–5022-S (Partec GmbH, Germany), following method of Doležel et al. [10]. Freshly harvested leaf tissue weighing 25 gm approximately equal to about 0.5 cm^2^ area, of both standard and sample species were chopped using a sharp razor blade in 500 µl of cold nuclei extraction buffer (NEB) in a petri dish. Samples were chopped quickly but not intensely to minimize the release of cytosolic compounds. 200 µl of CyStain UV Precise P staining buffer was combined with 12 µl of PI and 6 µl of RNase A to create the staining solution for each sample. The mixture was then gently shaken (Fig. 3). The chopped tissue in the buffer was homogenized by repeated pipetting and incubated for 60 s (Fig. 3). The resulting homogenate was poured into a 3.5 ml sample tube using a 50 µm nylon mesh filter (Partec CellTrics). The clear supernatant thus obtained was stained by adding 2.0 ml staining solution (CyStain UV Precise P) containing 12 µl PI (50 µg/ml) and 6 µl RNaseA (50 µg/ml). Stained samples were incubated on ice and kept in dark for 1 h before being analyzed. Nuclear DNA content was measured using CyFlow Cube 8 flow cytometer (Sysmex Partec GmbH, Germany), equipped with 532 nm laser light source and an orange-red fluorescence emission (>590 nm). Histograms were acquired using the CyFlow Cube 13 software (Fig. 3). Amplification settings of the flow cytometer were kept constant while analyzing the test sample and the internal reference or the standard sample. High-quality histograms were obtained by manually defining a gated area that included the fluorescence signals from PI-stained G0/G1 (quiescent phase/first growth phase) nuclei, free from cellular debris. The median fluorescence value was acquired using the CyFlow Cube 13 software. Further FCS express software (7.04.0018) was used for data representation. DNA 2C value of each sample was estimated by comparing the median PI fluorescence intensity (MFI) values of both sample and standard, using the following formula:

$$\text{Sample}\;2C\;\text{DNA}\;\text{Content}\;\left(\text{pg}\right)=\text{Reference}\;2C\;\text{value}\times \frac{\left(\text{Sample}\;2C\;\text{mean}\;\text{peak}\;\text{position}\right)}{\left(\text{Reference}\;2C\;\text{mean}\;\text{peak}\;\text{position}\right)}$$

## Limitations

Not applicable.

## Ethics Statement

The authors have read and followed the ethical requirements for publication in Data in Brief and confirmed that the current work does not involve human subjects, animal experiments, or any data collected from social media platforms.

## CRediT authorship contribution statement

**Jaya Arora:** Conceptualization, Methodology, Validation, Formal analysis, Investigation, Writing – original draft. **Suman Lakhanpaul:** Resources, Data curation, Supervision, Writing – review & editing. **Kumar Manish:** Writing – review & editing. **Maharaj K. Pandit:** Conceptualization, Supervision, Resources, Project administration, Writing – review & editing.

## Data Availability

Genome size estimation of Himalayan plants (Original data) (Mendeley Data).
